# Case report: Technical risk mitigation and management strategies of semi-implantable intrathecal drug delivery systems for advanced cancer pain: a case-based study

**DOI:** 10.3389/fonc.2026.1767460

**Published:** 2026-04-15

**Authors:** Yi Lu, Yujia Xie, Xinyao Ye, Fan Yang, Xinhua Yao, Jianbin Xiao, Guangfang Zhang, Chao Liu, Bao Wang

**Affiliations:** 1 Department of Anesthesiology, The Affiliated Traditional Chinese Medicine Hospital, Guangzhou Medical University, Guangzhou, China; 2 Graduate School, Guangzhou Medical University, Guangzhou, China; 3 Department of Anesthesiology, Guangdong Provincial Hospital of Chinese Medicine, Guangzhou, China; 4 Department of Anesthesiology, Guangzhou Twelfth People's Hospital, Guangzhou, China

**Keywords:** advanced cancer pain, catheter occlusion, multidisciplinary pain management, opioid withdrawal syndrome, palliative care, semi-implantable intrathecal drug delivery system

## Abstract

**Objective:**

This study investigated the clinical efficacy, technical risks, and management strategies of the semi-implantable intrathecal drug delivery system (IDDS) as a “fourth-step” analgesic regimen for refractory advanced cancer pain.

**Methods:**

We retrospectively analyzed three representative cases of patients with advanced cancer pain who underwent semi-implantable IDDS implantation under fluoroscopic guidance in a digital subtraction angiography (DSA) suite.The study was approved by the Institutional Review Board (IRB), and all patients provided written informed consent. Analgesic efficacy was assessed using the Visual Analog Scale (VAS) score. Technical challenges—including placement verification without cerebrospinal fluid (CSF) backflow, mechanical failures, and opioid dependency—were evaluated to develop standardized management protocols.

**Results:**

Significant analgesia was achieved in all cases. Preoperative VAS scores (8–10) decreased to 0–4 within 24 hours and stabilized at 0–1 after 72 hours. Specific technical risks were successfully mitigated through individualized strategies:

**Placement verification:**

In a cachectic patient (Case 1) with an iodine contrast allergy and absent CSF backflow, a “morphine test dose” (0.1 mg bolus) was utilized as functional confirmation of subarachnoid placement, justified by the rapid 10-minute analgesic onset.

**Mechanical issues:**

Repeated occlusion alarms in Case 2, caused by pressure sensitivity at the device’s minimum flow rate (0.1 mL/h), were resolved by diluting the drug concentration to increase the basal flow rate and implementing a “U-shaped” redundant catheter loop at the port site.

**Opioid withdrawal:**

Case 3 developed acute psychological withdrawal symptoms (COWS score: 14) following the rapid reduction of high-dose systemic opioids. This was managed through a multidisciplinary cross-titration protocol involving oral oxycodone and cognitive behavioral therapy. No infectious complications occurred during the follow-up period (up to 8 weeks).

**Conclusion:**

The semi-implantable IDDS serves as a crucial, cost-effective palliative option for patients with advanced cancer pain and a life expectancy of 3–6 months. While its externalized design introduces technical risks, these can be effectively managed through high-resolution fluoroscopy-guided placement, flow-rate optimization, and multidisciplinary support. Standardized troubleshooting algorithms and strict patient stratification are essential to achieve the goal of “pain-free survival.”

## 1 Introduction

In modern pain management, treating advanced cancer pain remains a formidable challenge. Although the World Health Organization (WHO) three-step analgesic ladder is widely adopted, approximately 20% of patients develop refractory pain, necessitating solutions that transcend conventional delivery routes (1–6). The intrathecal drug delivery system (IDDS) has emerged as a core “fourth-step” strategy for refractory pain by delivering analgesics directly into the spinal subarachnoid space. This method achieves potent analgesia with opioid doses as low as 1/300 of the oral equivalent, thereby significantly reducing systemic adverse effects (7–10).

The clinical application of IDDS requires balancing the characteristics of its two primary configurations. Fully implantable systems, with the pump embedded subcutaneously, offer a low risk of infection (<2%) and high patient comfort (11). However, their high cost (CNY 150,000–200,000) makes them most suitable for patients with adequate financial means and a life expectancy exceeding six months (12); while their drug reservoir capacity is smaller (typically ≤40 mL), this is easily managed through routine percutaneous refills (12). Conversely, the semi-implantable IDDS features an externalized drug reservoir and catheter, offering significant advantages such as lower cost (CNY 10,000–20,000), a large reservoir capacity (up to 300 mL), and flexible drug compatibility. Consequently, it serves as a cost-effective palliative option for patients with a life expectancy of 3–6 months (13). Nevertheless, its externalized design significantly elevates the risk of infection (reported rates: 5–8%), catheter occlusion, and migration, demanding higher surgical expertise and multidisciplinary collaboration (14, 15).

Current clinical practice with semi-implantable IDDS reveals a critical paradox. While these systems provide rapid and profound analgesia, the technical challenges—including difficult catheter positioning in cachectic patients, mechanical failures, and the management of opioid dependency—lack standardized management protocols. Existing research predominantly focuses on fully implantable systems, resulting in a scarcity of systematic analyses regarding risk stratification for semi-implantable configurations in real-world settings. Based on three representative cases that encapsulate these specific hurdles, this paper provides an in-depth analysis of clinical efficacy, technical risks, and corresponding management strategies. This study aims to furnish an evidence-based foundation for optimizing the safe application of cost-effective IDDS and to bridge the existing evidence gap in advanced cancer palliative care.

## 2 Case series

### 2.1 General surgical procedure

To ensure technical reproducibility and reduce descriptive redundancy, all implantations were performed using a standardized protocol in a Digital Subtraction Angiography (DSA) suite (operated in fluoroscopy mode) for superior spatial resolution (**Table 1**).

**Table 1:** Standardized baseline and pre-operative clinical status.

Case	Diagnosis (stage/metastases)	Pain phenotype/site	Pre-IDDS OMEDD (mg/d)	Pre-op VAS	ECOG score	Basis for life expectancy
1	Nasopharyngeal Ca (IV/Bone, Lung)	Mixed (Lumbosacral)	240	10	3	Multiple organ failure
2	Appendiceal Mucinous Ca (IV/Omental)	Somatic (Abdominal)	450	8	3	Extensive omental metastases
3	Pancreatic Head Ca (IV/Liver)	Neuropathic/Visceral	360	10	4	Liver failure/Cachexia

OMEDD, Oral Morphine Equivalent Daily Dose; VAS, Visual Analog Scale; ECOG, Eastern Cooperative Oncology Group.

Implanted Component: The ZS2 Series Implantable Device (Model: ZS2-I-Q-1.1/0.7-1000; Beijing Yuetong Medical Device Co., Ltd.) was used, featuring a kink-resistant polyurethane intrathecal catheter.

External Component: A portable electronic infusion pump (Model: ZNB-II/IIC; Jiangsu Annuo Medical Technology Co., Ltd.) was utilized with a 300 mL medication reservoir.

Method: Under local anesthesia, a 15G Tuohy needle was targeted at the L2/3 interspinous space. The catheter was advanced to the T7–T9 level. A subcutaneous pocket was created for the titanium-alloy port, which was then connected to the external pump via a non-coring Huber needle.

### 2.2 Case 1: difficult placement in a cachectic patient

Case Presentation: A 49-year-old male with Stage IV nasopharyngeal carcinoma presented with refractory mixed lumbosacral pain (Pre-operative OMEDD: 240 mg/day; VAS: 10). Physical examination revealed severe cachexia and multiple organ metastases.

**Figure 1:**
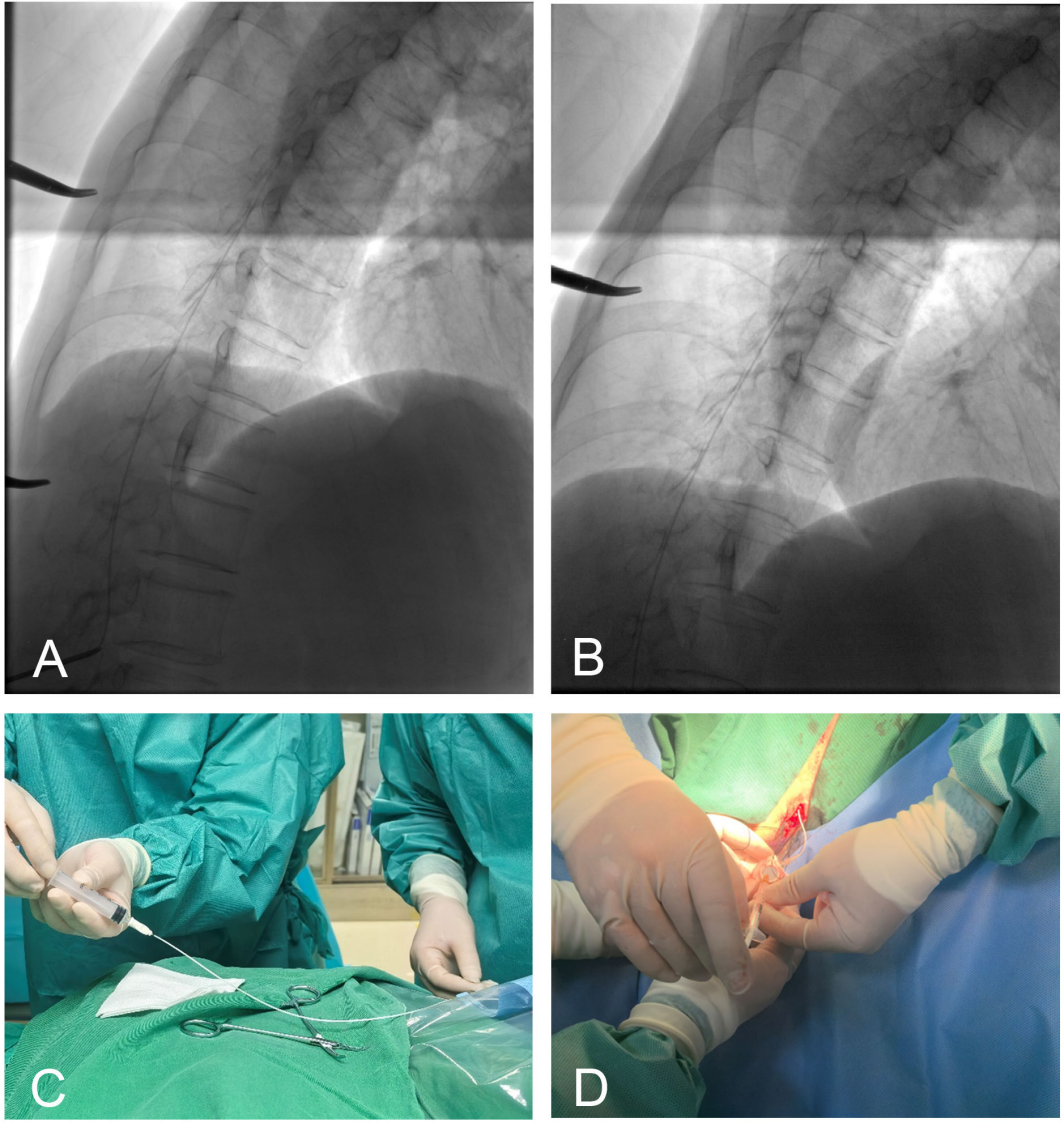
**(A)** Needle insertion at the puncture site. **(B)** Fluoroscopic confirmation of catheter tip placement at the T7–8 vertebral level. **(C)** Absence of cerebrospinal fluid backflow upon aspiration. **(D)** Marked relief of cancer pain following administration of a 0.1 mg morphine bolus via the pump.

Technical Challenge and Justification: During the procedure, successful needle advancement failed to yield cerebrospinal fluid (CSF) backflow (**Figures 1A–C**). This was attributed to a collapsed subarachnoid space and extremely low CSF pressure secondary to extreme cachexia. Contrast-enhanced fluoroscopy was contraindicated due to the patient’s documented hypersensitivity to iodinated contrast media and the risk of nerve root irritation in a narrowed space.

Management: A “morphine test dose” of 0.1 mg was administered via the catheter (**Figure 1D**). The patient reported profound analgesia (VAS decreased to 3 within 10 minutes). This rapid onset—characteristic of intrathecal μ-opioid receptor activation—functionally confirmed subarachnoid placement.

Dosing and Outcomes: The reservoir contained 0.33 mg/mL morphine and 1.0 mg/mL ropivacaine. Postoperative settings and outcomes are detailed in **Table 2** and **3**. The patient achieved a VAS of 0–1 by day 3 and was followed until death 8 weeks postoperatively due to malignancy progression.

**Table 2:** Standardized intrathecal dosing regimens and settings at discharge.

Case	Morphine conc. (mg/mL)	Ropivacaine conc. (mg/mL)	Basal rate (mL/h)	Basal morphine (mg/day)	Basal ropivacaine (mg/day)	PCA bolus (Morphine mg)	Lockout interval (min)
1	0.33	1.00	0.1	0.80	2.40	0.033	15
2*	0.27	0.67	0.5	3.24	8.04	0.135	15
3	0.11	0.50	0.3	0.80	3.60	0.011	20

Case 2: Settings reflect the optimized regimen after drug dilution to resolve persistent occlusion alarms. The basal rate was increased from the initial 0.1 mL/h (which triggered pressure-sensitive alarms due to micro-fluctuations) to 0.5 mL/h to maintain consistent intraluminal pressure while keeping the therapeutic dose stable. Case 3: In addition to the intrathecal regimen, the patient was managed with an oral oxycodone bridge (40 mg/day; equivalent to 60 mg OMEDD) during the cross-titration phase to mitigate acute physiological withdrawal symptoms. Safety Monitoring: All patients were monitored every 4 hours for respiratory rate, sedation (SASS score), and motor function. No adverse events or local anesthetic toxicity were recorded at these dosages. Conc., concentration; mg/day, milligrams per day; mL/h, milliliters per hour; PCA, Patient-controlled analgesia (bolus volume: 0.1 mL); *PCA bolus volume for Case 2 was 0.5 mL to maintain consistency after dilution. OMEDD, Oral morphine equivalent daily dose.

**Table 3:** Summary of longitudinal outcomes and follow-up.

Case	Baseline VAS	24h Post-op VAS	Day 3 VAS	Day 7/discharge VAS	Total follow-up	Endpoint/reason
1	10	3	0–1	0–1	8 weeks	Death (Organ Failure)
2	8	4	0–1	0–1	1 week	Discharge (In-hospital focus)
3	10	3	0–1	0–1	1 week	Discharge (In-hospital focus)

Outcome assessment focused on VAS reduction and subjective sleep quality. The lack of long-term follow-up for Cases 2 and 3 is a recognized limitation of this retrospective series.

### 2.3 Case 2: mechanical challenges and occlusion management

Case Presentation: A 65-year-old woman with Stage IV appendiceal adenocarcinoma presented with refractory somatic abdominal pain (Pre-operative OMEDD: 450 mg/day; VAS: 8). The patient exhibited severe cachexia and markedly loose skin.

**Figure 2:**
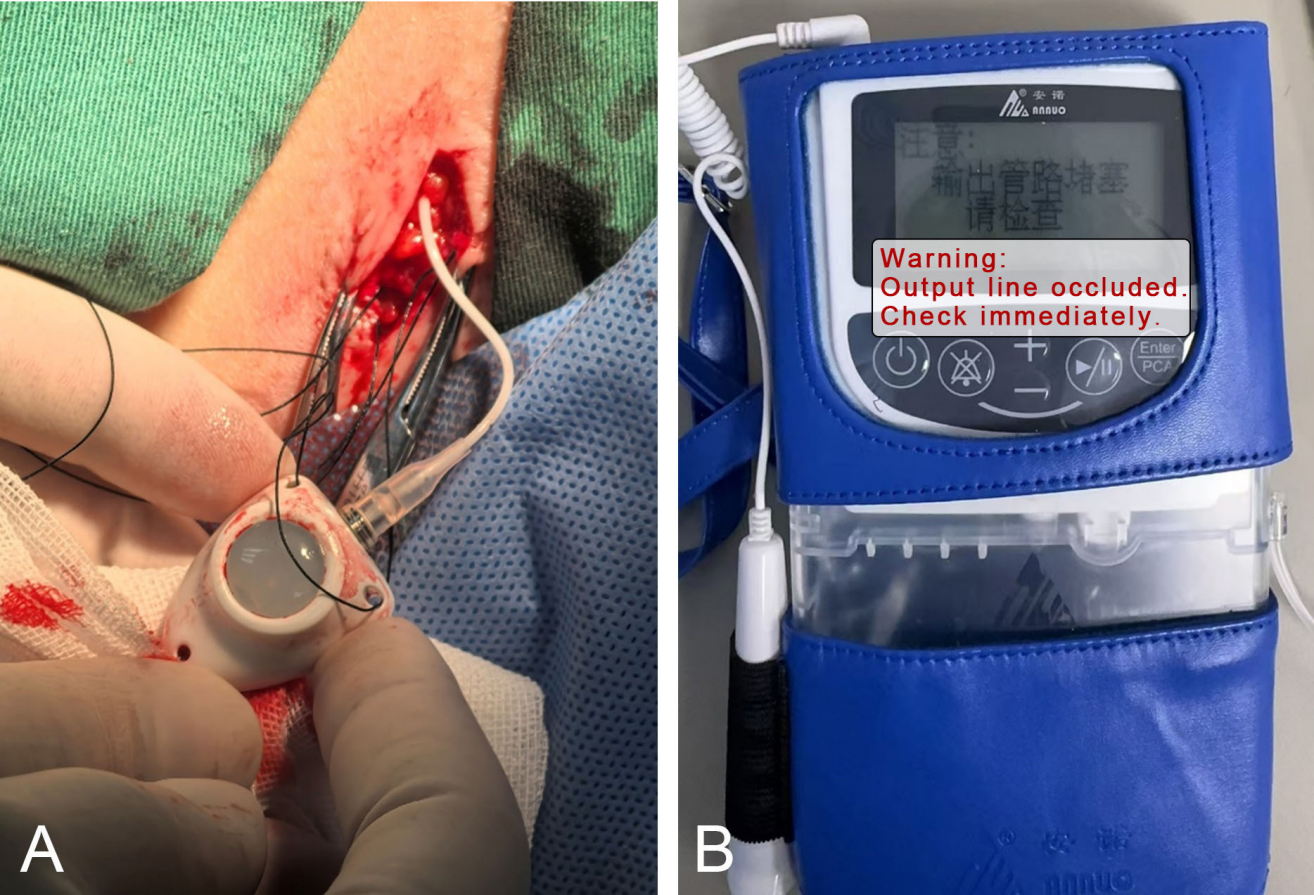
**(A)** Port fixation using the three-suture technique. **(B)** Pump alarm indicating catheter occlusion.

Technical Challenge: Implantation followed the standardized fluoroscopy-guided protocol, and cerebrospinal fluid (CSF) backflow was successfully obtained. Due to the patient’s loose skin, a three-suture technique was required to anchor the port to the fascia to prevent migration (**Figure 2A**). However, this tight fixation precluded the creation of a “U-shaped” redundant catheter loop (buffer) at the port site. One hour postoperatively, the system triggered repeated “occlusion alarms” (**Figure 2B**) despite no obvious external tubing kinks.

Troubleshooting Logic: As standardized in **Table 4**, port patency was confirmed via a syringe aspiration test (ruling out fibrin sheath or port-needle displacement), and catheter migration was ruled out via radiography. Technical analysis revealed that the initial basal rate of 0.1 mL/h (equivalent to 3.24 mg/day morphine based on the initial concentration of 1.33 mg/mL) made the system hypersensitive to intraluminal pressure fluctuations. In the absence of a redundant buffer loop, minor positional shifts increased resistance beyond the pump’s pressure threshold.

**Table 4:** Troubleshooting pathway for IDDS occlusion alarms.

Step	Action	Objective
1	Syringe Aspiration Test	Verify port patency and needle position
2	Radiographic Imaging	Rule out catheter kinking or tip migration
3	Flow Rate Optimization	Dilute drug to increase basal rate above 0.2 mL/h
4	Surgical Buffer	Implement “U-shaped loop” and elastic binder

Management: To stabilize the system, the medication was diluted. The basal rate was increased to 0.5 mL/h while maintaining the therapeutic dose at 3.24 mg/day morphine (and 8.0 mg/day ropivacaine). This adjustment provided consistent hydraulic pressure and effectively eliminated the alarms. The patient achieved a VAS score of 0–1 and was discharged on postoperative day 7 following suture removal.

### 2.4 Case 3: management of opioid withdrawal syndrome

Case Presentation: A 62-year-old male with Stage IV pancreatic head malignancy presented with intense visceral abdominal pain (Pre-operative OMEDD: 360 mg/day; VAS: 10). Despite high-dose systemic opioids (oxycodone and fentanyl patches), the pain remained refractory and was exacerbated by meals. Physical examination and imaging confirmed hepatic metastases and severe cachexia.

**Figure 3:**
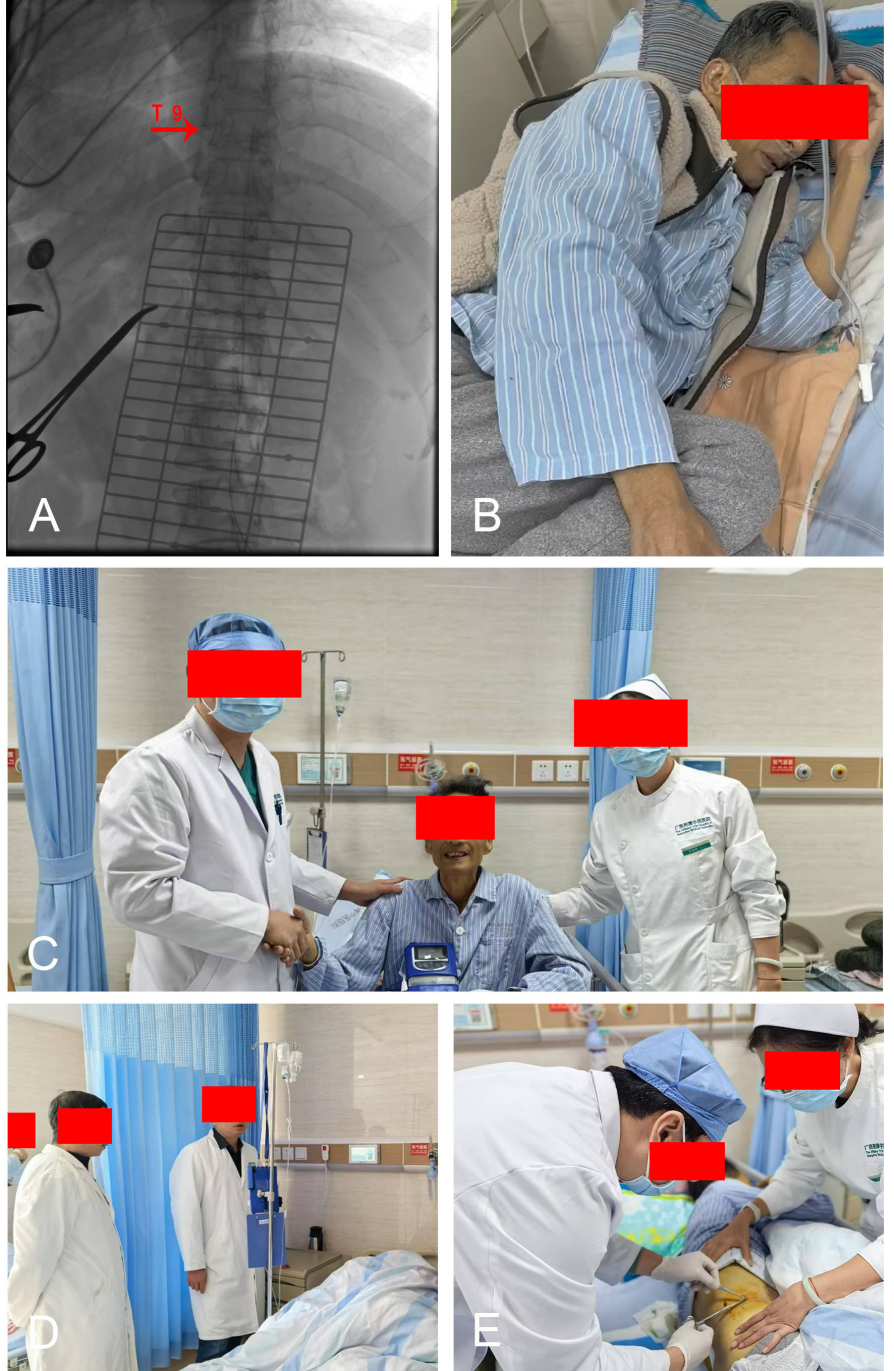
**(A)** Catheter position was confirmed at the T9 level. **(B)** Comparison of Preoperative and **(C)** Postoperative Status. **(D)** Assessment of Drug Dependence Severity and Psychological Status. **(E)** Sutures were removed on postoperative day 7.

Treatment Course: A semi-implantable IDDS was implanted using the standardized fluoroscopy-guided protocol. CSF backflow was successfully obtained at the L2/3 level, and the catheter tip was positioned at T9 (**Figure 3A**). Total blood loss was 3 mL. Initial intrathecal settings provided a basal dose of 0.80 mg/day morphine and 3.60 mg/day ropivacaine (**Table 2**).

Withdrawal Management: Postoperatively, somatic pain was effectively controlled (VAS 0–1 by day 1) (**Figures 3B, C**). However, on day 2, despite the absence of physical pain, the patient developed lacrimation, rhinorrhea, and severe psychological distress. A multidisciplinary assessment yielded a Clinical Opiate Withdrawal Scale (COWS) score of 14, diagnosing acute opioid withdrawal syndrome precipitated by the rapid reduction of high-dose systemic opioids (**Figure 3D**).

Management: A “cross-titration” protocol was initiated to manage the physiological dependence. Oral oxycodone was prescribed at 20 mg twice daily (total 40 mg/day; equivalent to 60 mg OMEDD) as a temporary bridge, supplemented by cognitive behavioral therapy (CBT) to support psychological stabilization. Withdrawal symptoms resolved by day 5. The patient was discharged on day 7 (**Figure 3E**) with optimized intrathecal settings and a plan for the gradual tapering of the oral bridge medication.

## 3 Discussion

The intrathecal drug delivery system (IDDS) serves as a critical “fourth-step” strategy for refractory cancer pain. Clinical decision-making requires a balance between two primary configurations. Fully implantable systems offer a lower infection risk (<2%) but are often inaccessible due to high cost (CNY 150,000–200,000) (16).

In contrast, the semi-implantable IDDS is a cost-effective alternative (CNY 10,000–20,000) featuring a larger drug reservoir (up to 300 mL) (17). These characteristics make it a viable palliative bridge for patients with a life expectancy of 3–6 months (18, 19). However, as shown in our series, the externalized interface necessitates specialized management of mechanical and physiological risks.

## 4 Clinical role and positioning

The core value of IDDS lies in achieving potent analgesia at roughly 1/300th of the oral opioid dose by targeting the spinal dorsal horn directly (20). In this series, all patients achieved a VAS of 0–1 within 72 hours, significantly reducing systemic adverse effects such as sedation and constipation (21–23). This positioning allows terminal patients to achieve “pain-free survival” and improved social reintegration during their remaining months.

Safety Monitoring Protocol (Applied to all cases): Postoperative safety surveillance included assessing vitals, respiratory rate, oxygen saturation (SpO2), and lower limb motor strength every 4 hours. No instances of respiratory depression, hypotension, or local anesthetic toxicity were observed in this cohort.

## 5 Technical risks and clinical challenges: a case-based analysis

The externalized components of the semi-implantable IDDS introduce specific risks that require a stratified management approach.

### 5.1 Procedure-related risks

#### 5.1.1 Difficult catheter positioning and verification (case 1)

In patients with extreme cachexia, narrowed intervertebral spaces and reduced epidural fat often lead to an “atypical loss of resistance” and “failure to obtain CSF backflow” due to low subarachnoid pressure. In Case 1, although high-resolution fluoroscopy confirmed the catheter’s vertebral level, subarachnoid placement could not be verified by aspiration.Contrast injection was avoided due to the patient’s documented hypersensitivity to iodinated contrast media and the risk of inducing severe headaches or nerve root irritation in a collapsed, low-pressure subarachnoid space.

Risk Mitigation: In such scenarios, we utilized a “morphine test dose” (0.1 mg bolus) as a functional verification tool. Intrathecal opioids target the *μ*-receptors in the dorsal horn with a rapid onset (5–15 minutes) (24), whereas epidural administration requires diffusion across the dura, resulting in significantly slower onset (30–60 minutes) and lower potency (25). A rapid analgesic response (within 10 minutes) distinguishes intrathecal placement. While CT-guided guidance is a valid alternative, the “analgesic test” remains a practical bedside confirmation when imaging remains ambiguous.

#### 5.1.2 Catheter securement and mechanical troubleshooting (case 2)

Patients with significant weight loss and loose skin are at high risk for port migration and catheter kinking (26). In Case 2, the “three-suture technique” stabilized the port but limited the space for a redundant catheter loop, leading to occlusion alarms at the device’s minimum basal rate (0.1 mL/h).

Troubleshooting Algorithm for Occlusion Alarms:

To standardize management, we recommend the following structured pathway: (i) Clinical Assessment: Perform a port aspiration test using a non-coring needle to rule out needle displacement. (ii) Imaging Review: Utilize lateral X-rays to assess for gross kinking or migration. (iii) Flow Rate Optimization: At the device’s minimum programmable rate (0.1 mL/h), the system is hypersensitive to micro-fluctuations in intraluminal pressure. Diluting the drug concentration to allow an increase in basal rate to ≥0.5 mL/h maintains more consistent hydraulic pressure and reduces false-positive alarms. (iv) Surgical Revision: If alarms persist, intraoperatively create a “U-shaped redundant loop” within the subcutaneous tunnel to serve as a buffer against positional traction.

### 5.2 Medication management risks

#### 5.2.1 Management of opioid withdrawal syndrome (case 3)

Patients on long-term high-dose systemic opioids are at significant risk for withdrawal when transitioning to intrathecal delivery (27, 28). In Case 3, despite a VAS score of 0–1, the patient exhibited lacrimation and rhinorrhea with a Clinical Opiate Withdrawal Scale (COWS) score of 14, indicating acute physiological withdrawal.

Clinical Management Protocol: (i) Preoperative Risk Stratification: Utilize standardized tools such as the DAST-10 to assess addiction risk. (ii) Cross-Titration Protocol: Avoid abrupt discontinuation of systemic opioids. Gradually taper oral/transdermal doses while titrating the intrathecal dose to roughly 1/300th of the oral equivalent. (iii) Multidisciplinary Intervention: Engage psychiatric services early for cognitive behavioral therapy (CBT) and pharmacological support (e.g., oral oxycodone bridge) to manage the transition from systemic to localized delivery.

#### 5.2.2 Technical limitation of low-flow infusion (case 2)

The minimum programmable flow rate for many intrathecal pumps (0.1 mL/h) is highly susceptible to intraluminal pressure fluctuations (29). In Case 2, increased resistance at this threshold triggered repeated false-positive occlusion alarms.

Optimization Strategies:

Rate Adjustment: Dilute the drug concentration (e.g., as performed in Case 2) to increase the basal infusion rate to ≥0.5 mL/h. Higher flow rates maintain more consistent hydraulic pressure within the catheter, reducing the likelihood of pressure-threshold triggers.

Material Selection: Preferentially select kink-resistant polyurethane catheters (e.g., the ZS2 series used in this study) over traditional silicone. Polyurethane offers higher tensile strength and structural integrity (30, 31), which is crucial for preventing micro-kinking in patients with significant weight loss.

Alarm Configuration: Enable the “pressure threshold alarm” function during the initial titration phase to detect mechanical compromise early before significant analgesia failure occurs.

### 5.3 Infection and maintenance-related risks

Infection remains the most critical complication for semi-implantable IDDS due to the externalized interface. While fully implantable systems carry a <2% infection risk, literature reports rates of 5–8% for semi-implantable configurations (**Table 5**) (32). These range from superficial surgical site redness to life-threatening spinal meningitis (33).

**Table 5:** Comparison of IDDS configuration risks and costs.

Feature	Semi-Implantable IDDS	Fully Implantable IDDS
Cost (Estimated)	CNY 10,000–20,000	CNY 150,000–200,000
Reservoir Capacity	Up to 300 mL (Annuo reservoir)	Typically ≤40 mL (refillable as needed)
Infection Risk	5–8% (externalized interface) (21)	<2% (completely subcutaneous)
Life Expectancy Suitability	3–6 months (Palliative)	>6 months (Chronic management)
Maintenance Complexity	High (Dressing changes/external care)	Low (Refills every 1–3 months)

Preventive and Control Measures: (i) Strict Asepsis: Implement rigid sterile protocols during weekly dressing changes and Huber needle access. (ii) Caregiver Training: Educate families on waterproofing and preventing traction on the external catheter. (iii) Early Explantation: If deep pump-pocket or catheter-tunnel infection is confirmed, surgical removal of the system is mandatory to prevent subarachnoid spread (34). No infectious complications occurred in our cohort under these strict maintenance protocols.

## 6 Optimizing clinical decision-making: patient stratification

**Figure 4:**
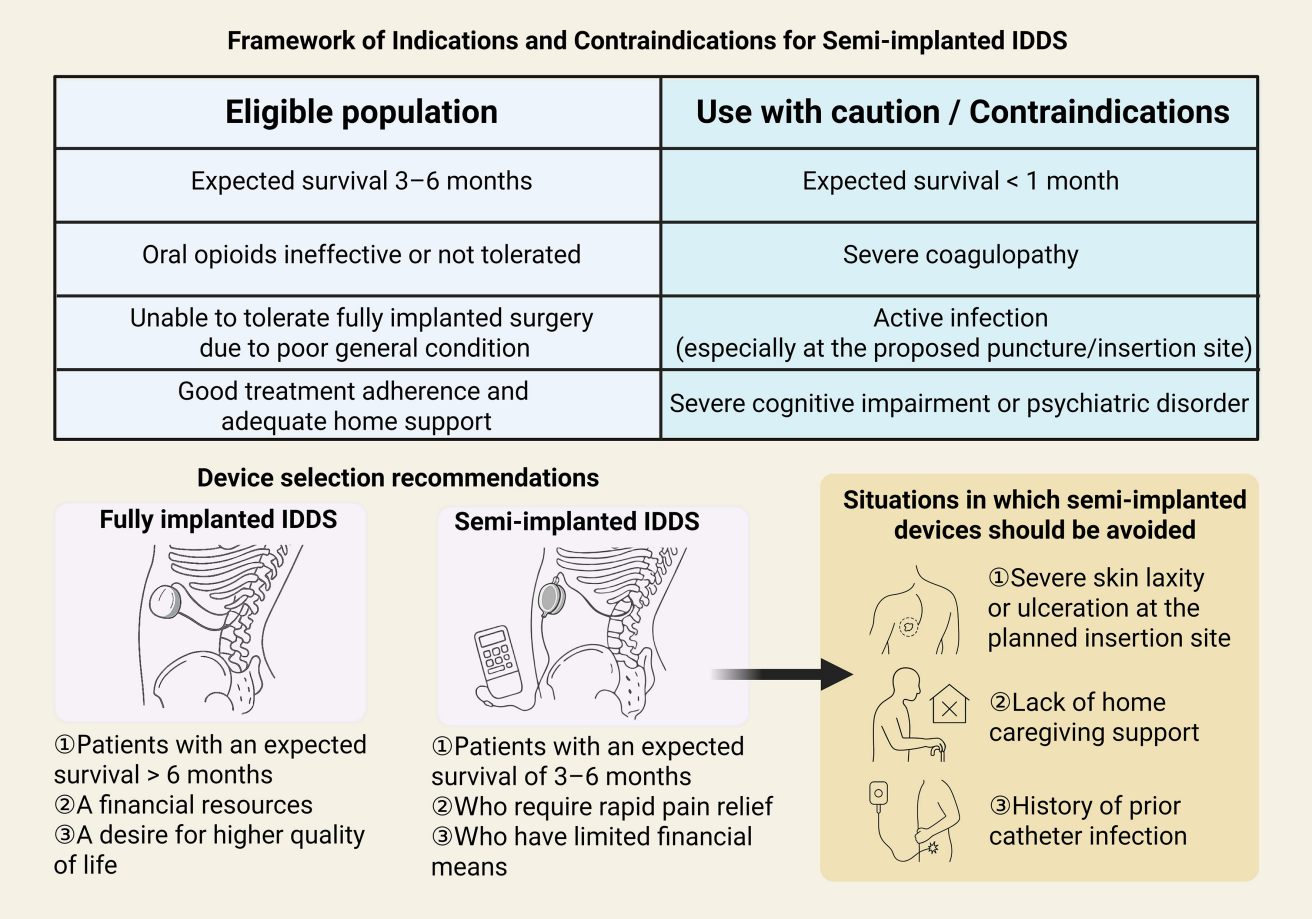
A clinical framework and device selection guide for semi-implantable IDDS.

Based on our findings, we propose a framework for patient selection (**Figure 4**). While life expectancy is a primary criterion, it must be balanced against the clinical team’s ability to provide multidisciplinary maintenance. We recommend semi-implantable IDDS specifically for patients with a life expectancy of 3–6 months who have failed WHO Step 3 interventions and have limited financial resources.

## 6.1 Study limitations

Although this study was conducted within a prospective clinical framework, several limitations should be acknowledged. First, the small sample size (n=3 representative cases) limits the generalizability of the troubleshooting algorithms and management protocols across a broader and more diverse patient population. While these cases were intentionally selected to illustrate specific technical hurdles, they may not encompass every possible clinical scenario. Second, the follow-up duration for Cases 2 and 3 was relatively short, primarily focusing on the immediate postoperative phase and symptom stabilization. Consequently, long-term complications, such as late-onset catheter migration, cumulative infection risks over several months, or changes in medication tolerance, could not be comprehensively assessed. Finally, this study lacks a comparative control group (e.g., patients managed with fully implantable systems or traditional systemic opioids), which precludes a statistical evaluation of relative efficacy and safety. Future multi-center prospective studies with larger cohorts and extended follow-up periods are required to validate these technical risk mitigation strategies as universal clinical standards.

## 7 Conclusion

The semi-implantable IDDS represents an effective and cost-efficient “fourth-step” analgesic bridge for patients with refractory cancer pain, particularly those with a life expectancy of 3–6 months and limited financial resources. However, its externalized design necessitates rigorous adherence to optimized technical protocols—such as fluoroscopy-guided placement for precise verification and the implementation of “U-shaped” redundant loops to prevent mechanical failure.Furthermore, proactive multidisciplinary management is essential to mitigate medication-related risks, specifically opioid withdrawal syndrome. By integrating these technical refinements with standardized risk management, clinicians can safely provide potent analgesia, achieving the fundamental goal of “pain-free survival” for advanced cancer patients.

## Data Availability

The original contributions presented in the study are included in the article/supplementary material. Further inquiries can be directed to the corresponding authors.
